# An update on the CNS manifestations of neurofibromatosis type 2

**DOI:** 10.1007/s00401-019-02029-5

**Published:** 2019-06-04

**Authors:** Shannon Coy, Rumana Rashid, Anat Stemmer-Rachamimov, Sandro Santagata

**Affiliations:** 1 Division of Neuropathology, Department of Pathology, Brigham and Women’s Hospital, Hale Building for Transformative Medicine, BTM8002P, 60 Fenwood Road, Boston, MA 02115 USA; 2 Department of Pathology, Boston Children’s Hospital, Boston, MA USA; 3 Harvard Medical School, Boston, MA USA; 4 Laboratory for Systems Pharmacology, Harvard Program in Therapeutic Science, Boston, MA USA; 5 Department of Pathology, Massachusetts General Hospital, Boston, MA USA; 6 Ludwig Center at Harvard, Boston, MA USA

**Keywords:** Neurofibromatosis type 2, Neurofibromatosis type II, NF2, Epidemiology of familial tumor syndromes, Schwannomatosis, Merlin, Schwannomin, ERM (ezrin/radixin/moesin) family scaffolding, Vestibular schwannoma, Meningioma, Glioma, Ependymoma, Neurofibroma, Plexiform schwannoma, Intraneural schwannoma, Cellular schwannoma, Posterior subcapsular lenticular opacities, LZTR1, SH3PXD2A-HTRA1, Verocay body, SUFU, SMARCE1, SMARCB1, Meningioangiomatosis, Glial micro-hamartoma, Glial hamartia, Wishart, Gardner, Von Recklinghausen, Manchester (NIH) Criteria, Manchester Criteria, Schwannoma, Central neurofibromatosis, Neurofibromin 2, Acoustic neuroma

## Abstract

Neurofibromatosis type II (NF2) is a tumor predisposition syndrome characterized by the development of distinctive nervous system lesions. NF2 results from loss-of-function alterations in the *NF2* gene on chromosome 22, with resultant dysfunction of its protein product merlin. NF2 is most commonly associated with the development of bilateral vestibular schwannomas; however, patients also have a predisposition to development of other tumors including meningiomas, ependymomas, and peripheral, spinal, and cranial nerve schwannomas. Patients may also develop other characteristic manifestations such as ocular lesions, neuropathies, meningioangiomatosis, and glial hamartia. NF2 has a highly variable clinical course, with some patients exhibiting a severe phenotype and development of multiple tumors at an early age, while others may be nearly asymptomatic throughout their lifetime. Despite the high morbidity associated with NF2 in severe cases, management of NF2-associated lesions primarily consists of surgical resection and treatment of symptoms, and there are currently no FDA-approved systemic therapies that address the underlying biology of the syndrome. Refinements to the diagnostic criteria of NF2 have been proposed over time due to increasing understanding of clinical and molecular data. Large-population studies have demonstrated that some features such as the development of gliomas and neurofibromas, currently included as diagnostic criteria, may require further clarification and modification. Meanwhile, burgeoning insights into the molecular biology of NF2 have shed light on the etiology and highly variable severity of the disease and suggested numerous putative molecular targets for therapeutic intervention. Here, we review the clinicopathologic features of NF2, current understanding of the molecular biology of NF2, particularly with regard to central nervous system lesions, ongoing therapeutic studies, and avenues for further research.

## Introduction

The neurofibromatoses are a group of familial tumor predisposition syndromes characterized by the development of distinctive neoplastic and dysplastic lesions which predominantly affect the central and peripheral nervous systems [1]. The neurofibromatosis family of disorders may consist of up to eight related clinical entities [2], though the term is most often used to described three syndromes—neurofibromatosis type I (NF1), neurofibromatosis type II (NF2), and schwannomatosis. Patients with symptoms suggestive of neurofibromatosis have been described since antiquity [3]. However, NF1 was first delineated as a nosological entity by von Recklinghausen in 1882 [4], while a syndrome consistent with NF2 was first described by Wishart in 1822 [5].

NF1 and NF2 were considered to represent alternate presentations of a single syndrome by Harvey Cushing, and in the decades following the 1917 publication of *Tumors of the Nervus Acusticus and the Syndrome of the Cerebellopontine Angle*, neurofibromatosis cases were frequently given a unifying diagnosis of von Recklinghausen’s disease [6]. Nevertheless, it was recognized during this period that individual cases of von Recklinghausen’s disease exhibited distinctive distributions of pathology, with some characterized by predominantly peripheral neurocutaneous lesions such as plexiform neurofibromas (“peripheral neurofibromatosis”), while others were characterized by development of distinctive intracranial and spinal lesions including bilateral vestibular schwannomas (“central neurofibromatosis”) [7, 8]. In the late 1980s, genetic analyses clearly demonstrated linkage of neurofibromatosis cases to distinct regions on chromosomes 17 and 22, with linkage to each region strongly correlating with the distribution and type of lesions encountered [9, 10]. These findings strongly suggested that von Recklinghausen’s disease may comprise multiple clinically and molecularly distinct syndromes.

In 1987, a National Institutes of Health (NIH) neurofibromatosis working group delineated distinct diagnostic criteria for NF1 and NF2 based on genetic linkage and clinicopathologic analysis [11]. Schwannomatosis, which shows some clinical overlap with NF2 including development of multiple non-vestibular schwannomas, was subsequently defined as a distinct nosological entity in 2005 [12] and refined to include molecular diagnostic criteria in 2013 [13]. Additional variants of neurofibromatosis such as variant neurofibromatosis (NF4), segmental neurofibromatosis (NF5), and multiple café au lait syndrome (NF6) have been described; however, most patients with these diagnoses exhibit alterations in the *NF1* gene on chromosome 17, suggesting that they may represent variant presentations of NF1, possibly as a consequence of genetic mosaicism, rather than distinct syndromes [14].

NF1 and NF2 have been critical models in the study of cancer, with each yielding numerous insights into the biology of neoplasia and genetic inheritance. Each syndrome presents with a distinctive set of neoplastic manifestations amenable to clinical identification, pathologic classification, and epidemiologic and molecular analysis. In this review, we describe the manifestations of NF2 with an emphasis on central nervous system lesions, recent insights into the biology and pathology of NF2, and directions for further study.

## Neurofibromatosis type II

Neurofibromatosis type II (NF2), previously known as “central neurofibromatosis”, demonstrates a predilection for “central” intracranial and spinal lesions, most characteristically vestibular schwannomas. Hereditary development of vestibular schwannomas was first recognized by Feiler and Ward [15], with an autosomal dominant mode of transmission subsequently established by Gardner and Frazier [7]. Bilateral vestibular schwannomas are the traditional pathognomonic diagnostic feature of NF2. However, population-based studies revealed that such lesions do not develop in all NF2 patients, and they may be absent in approximately 41% of patients at the time of diagnosis necessitating the use of additional diagnostic criteria [16]. Other distinctive lesions frequently encountered in NF2 include multiple schwannomas of cranial, spinal, or peripheral nerves, meningiomas, ependymomas, and ocular lesions. While neurofibromas may be diagnosed in patients with NF2, they are not clearly associated with this syndrome despite the syndrome’s description as a ‘neurofibromatosis’, in contrast to NF1, and many of the reported neurofibromas likely represent misdiagnosed hybrid schwannoma/neurofibromas.

NF2 is caused by inactivating alterations in the *NF2* gene on chromosome 22q12.2. The 100-kb *NF2* gene is encoded by 17 exons, and at least ten isoforms resulting from alternative splicing have been described in humans [17, 18]. Alternative isoforms most frequently result from alterations in the C-terminal exons 16 and 17. NF2-associated tumors are thought to result when additional somatic genetic alterations in vulnerable cell populations result in bi-allelic loss of function of *NF2*, as per Knudson’s two-hit hypothesis. However, experimental models have suggested that *NF2* mutations alone may not be sufficient to promote tumorigenesis, and additional genetic alterations are likely required [19].

Historically, two clinical forms of NF2 have been described. The Wishart phenotype is a more aggressive form of the disease, in which patients develop multiple neoplasms under 20 years of age with rapid progression of lesions. Other patients may exhibit a milder phenotype with fewer slow-growing tumors which typically arise later in life, described as the Gardner phenotype. It is now recognized that this spectrum of severity depends largely on the type of alteration in the *NF2* gene. Patients with truncating alterations that inactivate *NF2* exhibit more severe disease, whereas patients with missense loss-of-function mutations typically have a milder disease course [20]. Presentation with non-vestibular tumors in early life may be a harbinger of more severe multi-tumor disease [21, 22].

Pathogenic *NF2* alterations have a nearly 100% penetrance. Approximately 50% of NF2 patients present with symptoms and/or neoplastic manifestations by the age of 20, and nearly all by the age of 60 [20]. Large-population-based analyses have suggested that germline mutations in *NF2* are present in approximately 1 in 25,000 individuals with no gender predilection. However, the prevalence of diagnosed disease has been estimated at approximately 1 in 50,000 individuals, suggesting that not all patients with *NF2* mutations are subsequently diagnosed with the disease, which may occur due to non-pathogenic mutations, poor medical access, subtle or late-onset signs and symptoms, or other factors [23, 24].

Approximately 50% of NF2 cases are suspected to result from hereditary transmission from a parent with NF2, while the remainder appear to be due to de novo mutations in patients with no known family history. An estimated 1/3 of patients with de novo *NF2* mutations are mosaic for these alterations, with somatic mutations presumably occurring early in development [25, 26]. Patients with mosaic disease may test negative for *NF2* mutations by peripheral blood sequencing, despite meeting diagnostic clinical criteria for NF2, due to absence of the alteration in hematopoietic progenitors. Mosaic patients often have less severe disease and may present with unilateral vestibular schwannomas or segmental disease [27]. Patients with germline *NF2* mutations have a 50% probability of passing the disease to their children, while mosaic patients have a reduced chance of further transmission to their offspring due to absence of alterations in the germ cells of some patients. However, if affected, the children of mosaic patients will typically develop a more severe form of the disease than that of the parent due to a greater number of affected cells following germline transmission [28].

## Molecular biology of neurofibromatosis type II

*NF2* encodes the 70-kDa protein merlin (also known as neurofibromin 2 or schwannomin), which is widely expressed at high levels during human embryonic development. In adults, the distribution of expression is more limited and high levels of merlin are predominantly seen in Schwann cells, meningeal cells, lens fiber cells, and nerve cells [29]. Merlin is an ERM (ezrin/radixin/moesin) family scaffolding protein of the protein 4.1 superfamily, which includes ezrin, radixin, moesin, talin, and some protein tyrosine phosphatases. Merlin principally functions to mediate interactions between actin filaments, intracellular signaling effectors, and membrane proteins. Of the splice variants that have been described, all isoforms of merlin have a conserved N-terminal FERM domain which localizes the protein to the plasma membrane, predominantly at adherens tight junctions [30]. The N-terminal FERM domain of merlin is highly conserved and interacts with proteins such as CD43, CD44, and ICAMs, while the C-terminus binds to actin filaments, possibly through an alternative actin-binding site. ERM proteins such as merlin exhibit self-regulation through binding of the head and tail of the protein, with folding and unfolding regulated by phosphorylation of the C-terminus. Despite its homology to other ERM proteins and interaction with similar binding proteins, merlin seems to act in opposition to these proteins with regard to cell adhesion and proliferation. Whereas ERM proteins are active in a phosphorylated state and inhibited by cell–cell adhesion, merlin inhibits proliferation in response to cell–cell or cell–matrix adhesion as a result of serine dephosphorylation. Accordingly, loss of merlin leads to defective contact growth inhibition [31].

**Fig. 1:**
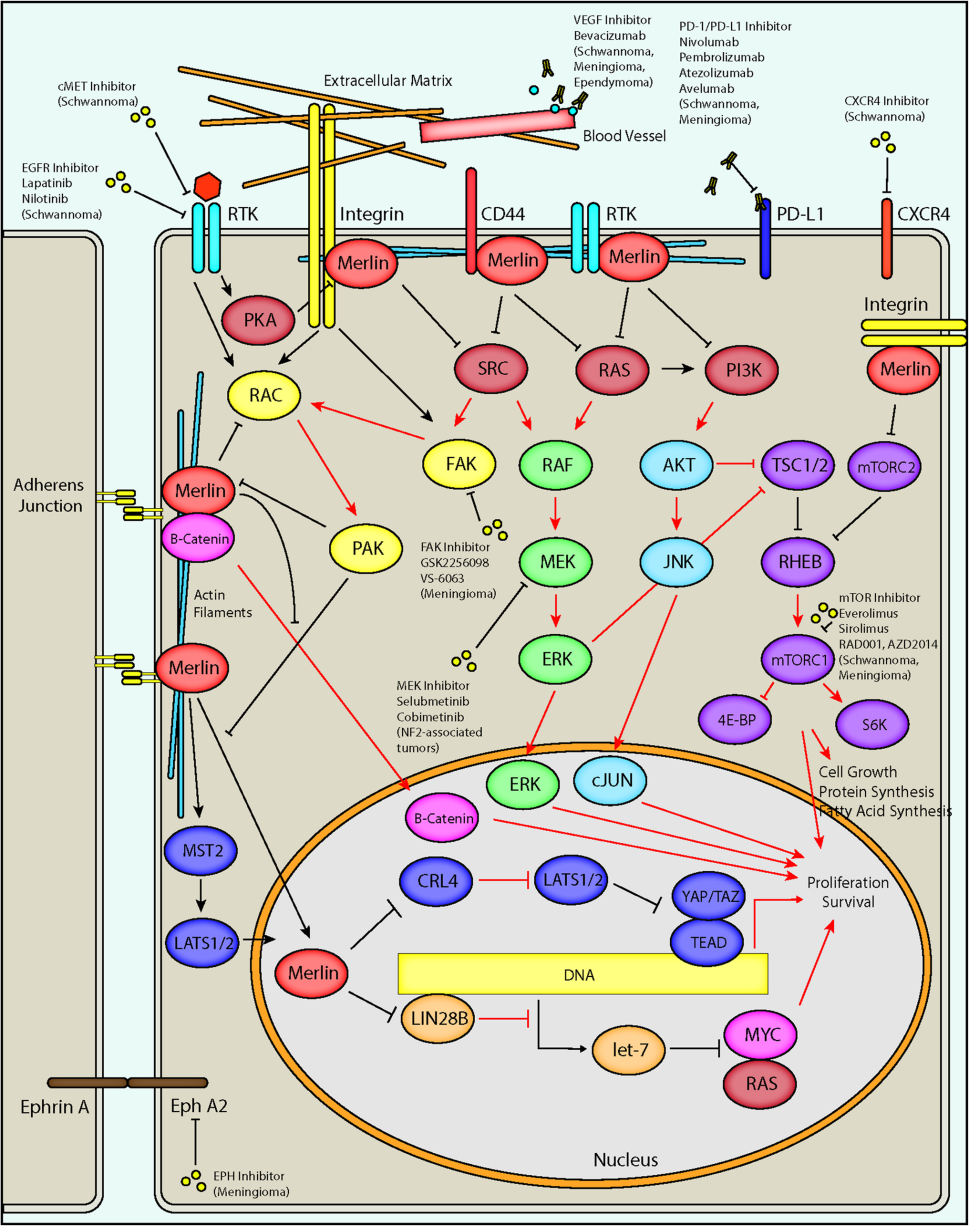
NF2/merlin signaling and potential therapeutic targets in NF2. A schematic diagram depicts major intracellular pathways regulated by merlin, the protein product of the *NF2* gene. Merlin is associated with the plasma membrane via its N-terminal FERM domain, where it interacts with adherens junctions and numerous cell surface receptors including integrins, receptor tyrosine kinases (RTK), and CD44. The C-terminal domain of merlin interacts with cytoskeletal actin filaments. Merlin has a predominantly inhibitory activity on downstream effectors including RAS, PI3K, RAC, and SRC, which may result in reduced downstream RAF/MEF/ERK, AKT/JNK/JUN, mTORC1, FAK, and RAC signaling. Merlin may inhibit translocation of β-catenin to the nucleus, reducing the output of canonical Wnt/β-catenin signaling. Merlin also interacts with the Hippo pathway by promoting MST1/2-mediated translocation of LATS1/2 to the nucleus as well as through inhibition of CRL4^DCAF1^, resulting in reduced transcriptional output of YAP/TAZ and TEA domain transcription factors (TEADs). In the nucleus, merlin may also inhibit the activity of LIN28A, thereby relieving inhibition of the *let*-*7* miRNA cluster leading to downregulation of proto-oncogenic proteins such as MYC and RAS. Loss of function of merlin via pathogenic mutations in NF2 patients results in alteration of downstream activity in each of these pathways (red arrows indicate steps with possible increased activity following loss of merlin function, as in NF2-associated tumors), potentially leading to increased cell growth, protein and fatty acid synthesis, proliferation, and survival. A variety of therapeutic avenues have been explored in NF2 patients, including inhibition of proteins regulated by merlin as well as other cellular receptors. Inhibition of the RAF/MEK/ERK pathway via small molecule MEK inhibitors is currently being explored in clinical trials for NF2-associated tumors (NCT02639546, NCT03095248). A phase 2 clinical trial is exploring small molecule inhibition of FAK by GSK2256098 (NCT02523014/A071401) in NF2 mutant meningiomas. Small molecule mTOR inhibitors including everolimus and sirolimus have shown therapeutic promise in NF2-associated schwannomas and meningiomas, and additional compounds including RAD001 and AZD2014 are in clinical trials for the treatment of progressive/symptomatic NF2-associated meningiomas (NCT02831257) and recurrent high-grade (WHO grade II/III) meningiomas (NCT03071874), respectively. The VEGF inhibitor bevacizumab has shown clinical utility in NF2-associated schwannomas, meningiomas, and ependymomas. Inhibition of the tyrosine kinase receptors cMET and EGFR may provide additional therapeutic opportunities in NF2-associated meningiomas. Additional therapeutic targets have been proposed, including the immunomodulatory PD-1/PD-L1 axis, the chemokine receptor CXCR4, and Ephrin receptor A2 (EphA2)

Merlin interacts with the components of numerous intracellular signaling pathways including Hippo, PKA, FAK/SRC, PI3K/AKT, Rac/PAK/JNK, WNT/B-catenin, integrins, receptor tyrosine kinase (RTK), Ras, MAPK, YAP, p21-activated kinase, CD44, and Rac/Rho [Fig. 1] [32–34]. In addition to its functions at the plasma membrane, merlin may translocate to the nucleus and suppress the E3 ubiquitin ligase CRL4^DCAF1^ [35, 36], and may also regulate protein and fatty acid syntheses via interactions with mTOR signaling [37]. Accordingly, loss of function of merlin has complex and distinct effects on intracellular signaling in different cellular lineages, and the precise mechanisms by which alterations in *NF2* cause the diverse manifestations of NF2 are not entirely clear. Given its well-described role in regulating cellular proliferation in response to adhesion via multiple pathways, loss of contact-mediated growth inhibition is hypothesized to play a principal role. However, increasing understanding of the functional effects of merlin loss on intracellular signaling pathways has led to the identification of multiple potential therapeutic avenues in NF2-associated tumors, including small molecule inhibition of mTOR, MEK, and FAK.

A wide variety of *NF2* alterations have been described in NF2 patients, including truncating, frameshift, nonsense, splice-site, and missense mutations, as well as structural alterations including insertions, deletions, and rearrangements. The most common alterations are splice-site mutations or nonsense mutations, most commonly in exons 1–8. Alterations may occur at a wide variety of different sites, though a few putative hotspots have been described including the exon 2 c.169C- > T nonsense mutation. Alterations in the conserved N-terminal FERM domain [38] and truncating mutations [39] are typically associated with the Wishart phenotype including younger age at diagnosis, a greater incidence of meningiomas, ophthalmologic and cutaneous lesions, and poor outcomes [40]. By contrast, missense and splice-site mutations, particularly in the 3′ end of the gene, are associated with the Gardner phenotype and a better prognosis with fewer meningiomas [41]. Increasing application of genomic and proteomic analyses in NF2 patients and other populations continues to expand our understanding of the molecular basis and diverse clinical manifestations of NF2, and may lead to additional insights into the biology and epidemiology of the disease.

## Diagnosis of neurofibromatosis type II

Diagnostic workup of NF2 is typically pursued in individuals judged to be at high risk for the disease, typically due to a family history of NF2 or clinical presentation with characteristic neoplastic or non-neoplastic manifestations. Clinical examination is often critical in making a correct and prompt diagnosis. Patients with NF2 may present with distinctive “plaque-like” cutaneous schwannomas, ophthalmologic findings, or neurologic deficits such as hearing loss or peripheral neuropathies which may prompt further radiologic or genetic evaluation. Café au lait spots may be present in up to 50% of patients but they are typically fewer in number and not associated with freckling as in NF1.

Specific criteria for the diagnosis of NF2 have been iteratively refined since the initial genetic delineation of the disease. The first criteria were established in 1987 by a United States National Institutes of Health (NIH) working group consensus statement [11]. This schema was amended (NIH 1991 and Manchester 1992) with the introduction of additional criteria, to include patients lacking bilateral vestibular schwannomas or a family history of NF2, but who were diagnosed with multiple schwannomas and/or meningiomas. These additional criteria increased the sensitivity of the diagnostic framework, and this system has been widely adopted (Table 1) [16].

**Table 1:** Manchester (NIH) Criteria (1992) for neurofibromatosis type II

Bilateral vestibular schwannomas *or*
Family history of NF2 *plus*
Unilateral vestibular schwannoma
Any two of: meningioma, glioma, neurofibroma, schwannoma, posterior subcapsular lenticular opacities
**Additional criteria**
Unilateral vestibular schwannoma *plus* any two of: meningioma, glioma, neurofibroma, schwannoma, and posterior subcapsular opacities
Multiple meningioma (two or more) *plus* unilateral vestibular schwannoma or any two of: glioma, neurofibroma, schwannoma, and cataract

Expanded criteria, aimed at identifying additional patients that do not have a family history of NF2 or vestibular schwannomas, were proposed in later classification systems (National Neurofibromatosis Foundation and Baser (1997)). More recently, revised criteria were proposed by Smith *et al.* in 2017 to exclude bilateral vestibular schwannomas occurring after 70 years of age, as such tumors are sporadic in up to 50% of patients in this age group without any associated stigmata of NF2 [42]. Additionally, incorporation of molecular criteria has been proposed, including detection of *NF2* gene variants in the peripheral blood or in multiple tumors, and exclusion of patients with lesions harboring mutations in *LZTR1* which are associated with schwannomatosis [43].

Further refinement of NF2 diagnostic criteria has been suggested following a recent study by Evans *et al.* in which nearly 3000 patients with molecular testing showed no evidence to support the inclusion of ‘glioma’ or ‘neurofibroma’ [44]. On the other hand, the inclusion of ‘ependymoma' in the diagnostic criteria of NF2 would potentially improve positive predictive value. In this study, for patients with unilateral vestibular schwannoma or multiple meningiomas, diagnosis of an ependymoma carried the highest positive predictive value for the diagnosis of NF2 of any lesion, with confirmed pathogenic mutations in up to 68% of patients. Further refinements may include exclusion of siblings with clearly uninvolved parents. These changes may improve the predictive value of the diagnostic criteria, help to better delineate NF2 and schwannomatosis, and allow for more effective clinical management of NF2 patients. However, to date no consensus has been reached for the refinement of diagnostic criteria, and the Manchester criteria remain the most widely used in clinical practice.

Continued epidemiologic analysis of large NF2 patient populations as well as the increasing availability of molecular data should result in further clarification of diagnostic criteria, particularly regarding delineation of related but separate syndromes such as schwannomatosis and the identification of NF2 patients with mosaic phenotypes, which remains challenging. To date, there are no published data on application of molecular biomarkers aside from *NF2* alterations in the diagnosis or management of NF2. However, efforts are underway to systematize sample and data collection, and to improve information sharing to accelerate these developments which should aid in further refinement of diagnostic criteria [45].

## Neoplastic manifestations of neurofibromatosis type II

### Schwannoma

Schwannomas are proliferations of neoplastic cells which exhibit histologic, ultrastructural, and molecular similarities to the Schwann cells of the peripheral nervous system. Schwannomas are typically benign slow-growing tumors characterized by a well-circumscribed population of spindled cells, though NF2-associated schwannomas may exhibit a variety of unusual features.

Vestibular schwannomas are the most common intracranial neoplasm in NF2 patients, affecting over 90% of individuals with the syndrome [46]. Vestibular schwannomas arise from the superior and inferior vestibular branches of the eighth cranial (vestibulocochlear) nerve with no definite predilection for either branch [47], though early studies incorrectly suggested a preference for the superior vestibular branch. Historically, vestibular schwannomas were often hypothesized to arise at the inner aspect of the internal auditory meatus in the Obersteiner–Redlich zone, which forms the junction of central and peripheral myelination. As with the historical classification of NF1 and NF2 as a single diagnosis (von Recklinghausen’s disease), this view was significantly supported and propagated by Harvey Cushing in his 1917 review of tumors of this region [6]. However, later anatomic studies showed that sporadic vestibular schwannomas frequently arise lateral to the central-peripheral junction and may arise anywhere along the course of the nerve, arguing against this theory [48]. More recently, a study of 41 pediatric NF2 patients with high-resolution MRI showed that many of the patients have numerous discrete tumor nodules along the course of the superior and inferior vestibular nerves [47]. Furthermore, Cre-mediated excision of the *NF2* gene under the control of the periostin promoter element in mice results in multiple discrete schwannoma tumor nodules along the eighth cranial nerve [49]. These findings collectively refute the hypothesis of a single point of origin in the transition zone and instead suggest that NF2-associated vestibular schwannomas are often comprised of numerous smaller lesions along the length of the nerve which have coalesced into a large multi-nodular mass, possibly explaining the greater aggressiveness and risk of recurrence of NF2-associated vestibular schwannomas. NF2 patients also frequently have additional nodules involving the cochlear nerve, labyrinth, and semicircular canals which may coalesce with lesions on the vestibular nerves and contribute to clinical symptoms [47]. The underlying cause for the predilection of formation of NF2-associated schwannomas along the branches of the vestibular nerve relative to other cranial nerves remains uncertain, and investigation of this question may provide interesting insights into the biology of NF2 and schwannomas.

**Fig. 2:**
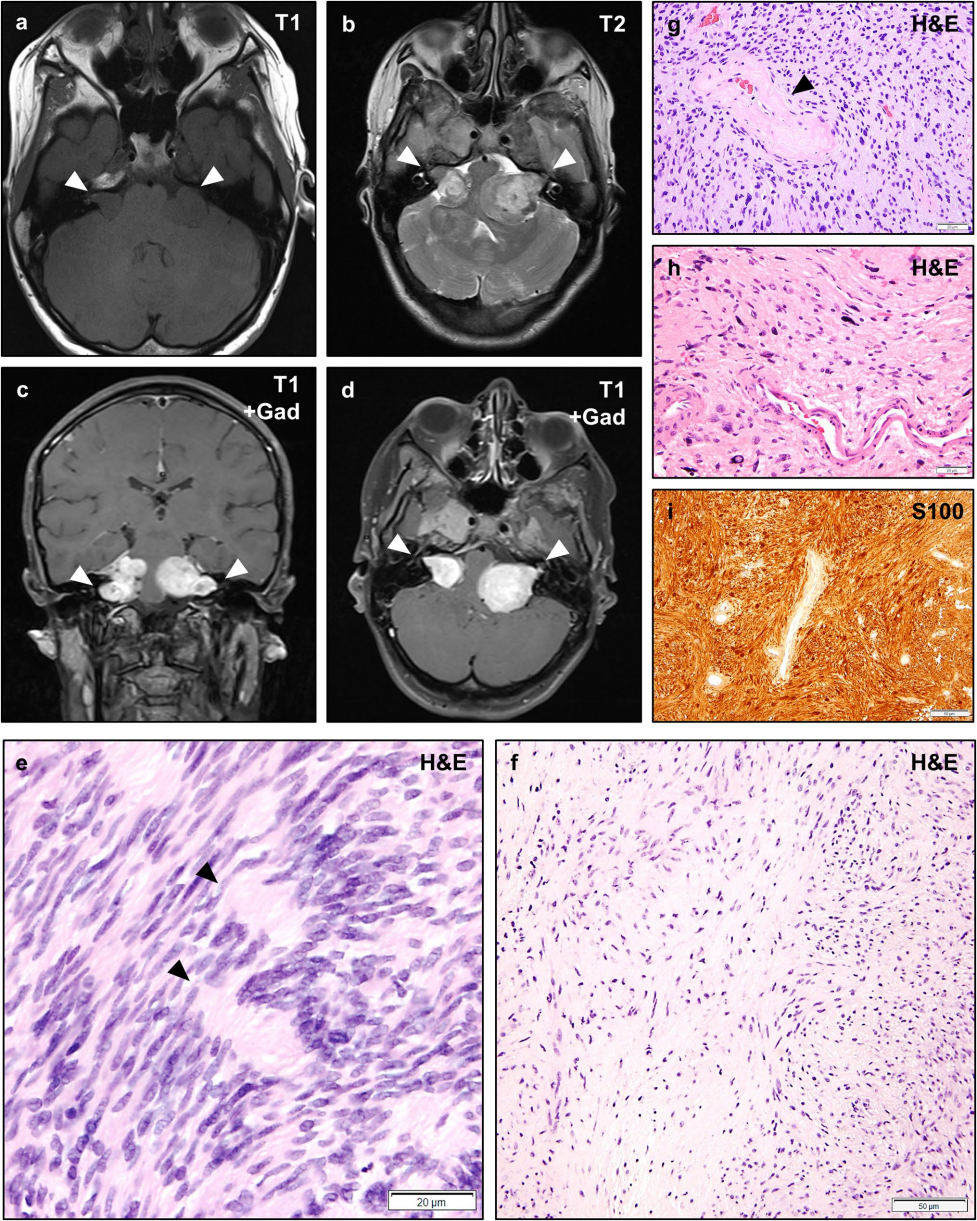
Vestibular Schwannomas in NF2. Magnetic resonance imaging (MRI) of an NF2 patient with bilateral vestibular schwannomas involving the cerebellopontine angle and internal acoustic meatus (**a**–**d**, arrowheads). Vestibular schwannomas are typically isointense on T1-weighted sequences (**a**), and show heterogeneous signal on T2-weighted sequences (**b**). Schwannomas typically exhibit intense gadolinium contrast enhancement (**c**–**d**). Histologically, schwannomas consist of spindled cells with tapering ends and eosinophilic to clear cytoplasm which may be architecturally arranged as densely cellular Antoni A regions with focally palisading nuclei that may form alternating layered hypercellular and eosinophilic paucicellular regions known as Verocay bodies (**e**, arrowheads), or less cellular Antoni B regions with more prominent extracellular matrix which may have a collagenized or myxoid appearance (**f**). Varying hypercellular and hypocellular regions may correlate with the heterogeneous intensity of schwannomas on T2-weighted sequences (**b**). Perivascular hyalinization is often a prominent histologic feature in schwannomas (**g**, arrowhead) and may aid in the diagnosis of lesions with an unusual histologic appearance. Schwannomas frequently exhibit degenerative changes (also known as “ancient change”) including increased pleomorphism, bizarre nuclei, and hyperchromasia (**h**). Such changes are not known to be associated with an increased risk of recurrence or malignant transformation. Schwannomas exhibit intense and diffuse cytoplasmic and nuclear staining with the S100 antigen (**i**). Scale bars 20 μm (**e**, **g**, **h**), 50 μm (**f**, **i**). A web-interactive tool for viewing images of NF2-related tumors is also available: http://tumoratlas.org/coy-acta-neuropathol-2019

Radiologically, schwannomas are typically isointense on T1-weighted MRI sequences (Fig. 2a), and heterogeneously intense on T2-weighted sequences (Fig. 2b). Schwannomas are typically brightly enhancing on post-gadolinium T1-weighted sequences (Fig. 2c, d). The presence of bilateral vestibular schwannomas is pathognomonic for the disease and a major diagnostic criterion for NF2 [11]. While NF2-associated vestibular schwannomas typically exhibit similar morphologic features as sporadic tumors with a predominance of cellular Antoni A regions which may exhibit palisading nuclei and Verocay bodies (Fig. 2e) and typically a lower proportion hypocellular Antoni B regions (Fig. 2f), they may have an increased mitotic index and higher risk of recurrence compared to sporadic lesions, possibly due to the polyclonal origin of some masses, difficulty with complete resection, and the presence of additional small nodules along the nerves. NF2-associated vestibular schwannomas may have a higher risk of malignant progression following radiation therapy, complicating treatment considerations [50]. Like other schwannomas, NF2-associated vestibular schwannomas frequently exhibit prominent perivascular hyalinization (Fig. 2g) and/or degenerative atypia (“ancient change”) (Fig. 2h) as well as strong and diffuse S100 staining by immunohistochemistry (Fig. 2i). Degenerative atypia is not known to be associated with more aggressive behavior or increased risk of recurrence, despite occasionally exhibiting an ominous histologic appearance. The biological basis of these changes and their consequences for cellular function are unclear, but investigation of this phenomenon may provide an interesting avenue for greater understanding of the basis of nuclear atypia in neoplasia and the mechanisms underlying nuclear structure.

**Fig. 3:**
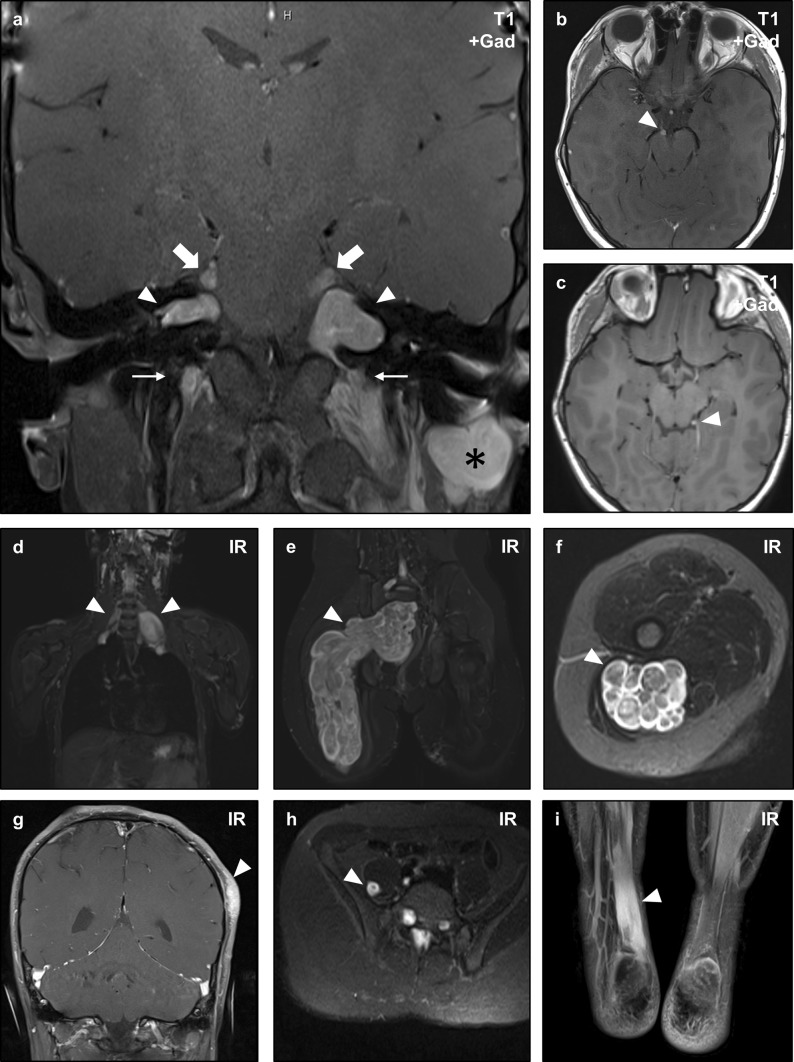
Non-vestibular schwannomas in NF2. Magnetic resonance imaging (MRI) of an NF2 patient with numerous intracranial and peripheral schwannomas. Coronal post-contrast T1-weighted sequences show bilateral vestibular (CNVIII) schwannomas (arrowheads) confirming the diagnosis of NF2, as well as bilateral trigeminal (CNV) nerve masses (large arrows), bilateral cervical nerve root masses (small arrows), and a post-auricular mass (asterisk) consistent with schwannomas (**a**). This patient also developed oculomotor (CNIII) schwannomas (**b**, arrowhead), and a small left trochlear nerve (CNIV) schwannoma (**c**, arrowhead). Whole body inversion recovery (IR) sequences showed large plexiform masses in the bilateral brachial plexus (**d**, arrowheads), and right sacral plexus (**e**, arrowhead). Axial slices of the latter mass demonstrate the multi-nodular plexiform architecture of the lesion (**f**, arrowhead). Schwannomas may also arise in unusual locations, as illustrated by a cutaneous lesion in the scalp (**g**, arrowhead), intramuscular lesion in the right iliopsoas muscle (**h**), and a diffusely infiltrative lesion in the right Achilles tendon (**i**, arrowhead) in this patient

**Fig. 4:**
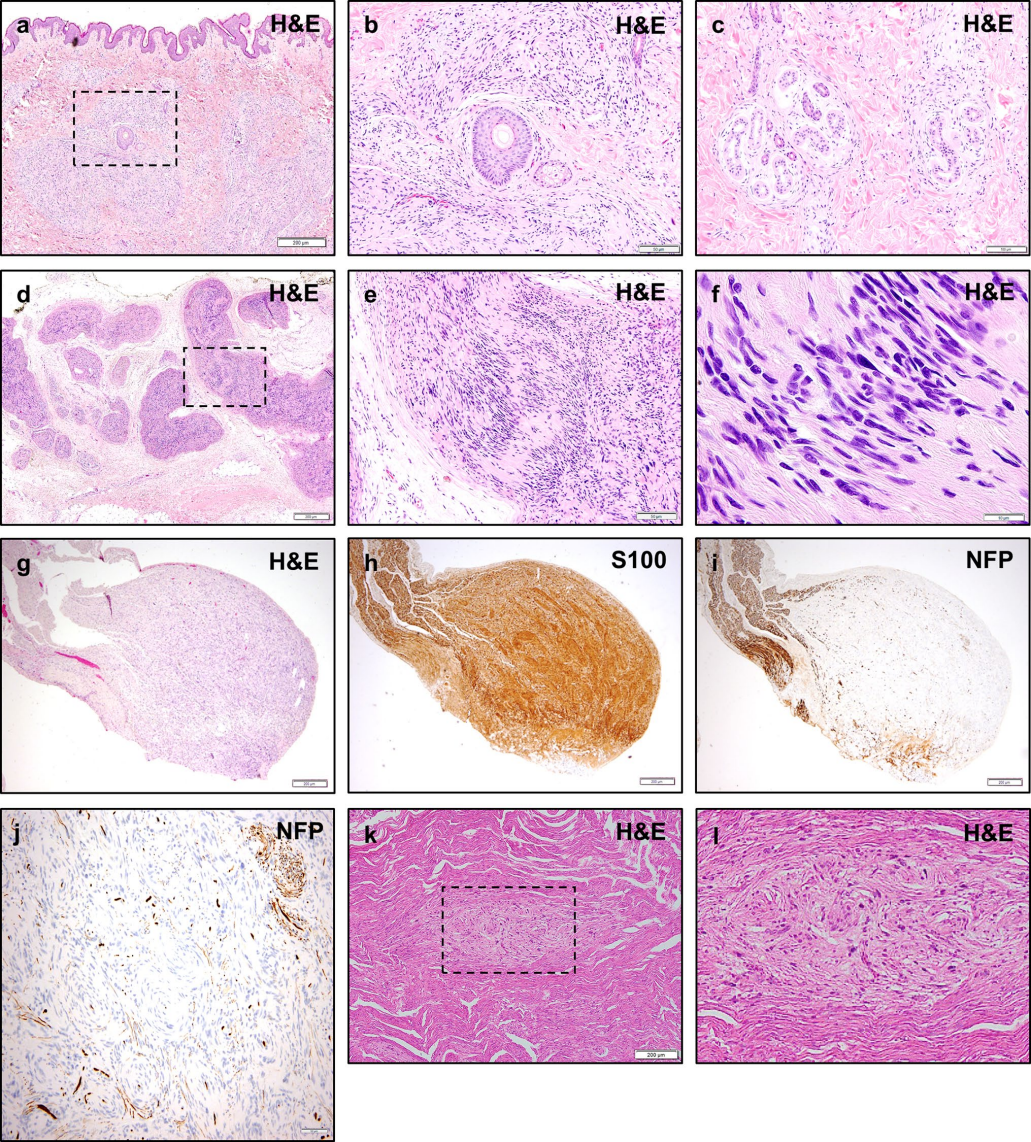
Histologic appearance of non-vestibular schwannomas in NF2. Non-vestibular peripheral schwannomas in NF2 patients may exhibit unusual histologic appearances. Skin biopsy of a cutaneous plaque-like plexiform schwannoma in an NF2 patient shows a nodular and infiltrative proliferation of neoplastic cells involving the dermis and subcutis (**a**). While sporadic schwannomas are typically circumscribed lesions with a pushing growth pattern, cutaneous schwannomas may infiltrate around skin adnexal structures such as hair follicles and sebaceous glands (**b**), and deeper eccrine glands and ducts (**c**). A plexiform schwannoma in the soft tissue of an NF2 patient shows multi-nodular growth along peripheral nerves (**d**), with typical morphologic features including palisading nuclei and Verocay bodies (**e**, **f**). NF2 patients may also develop intraneural schwannomas (**g**) with whorls of neoplastic cells infiltrating between individual nerve fibers, highlighted by S100 (**h**) and neurofilament protein (NFP) (**i**, **j**) immunohistochemistry. Schwann cell tumorlets may be identified in spinal nerve roots and peripheral nerves, and are hypothesized to represent precursor lesions to larger solitary or plexiform schwannomas (**k**, **l**). Scale bars 200 μm (**a**, **d**, **g**, **h**, **i**, **k**), 100 μm (**c**), 50 μm (**b**, **e**, **j**), 10 μm (**f**)

Schwannomas involving non-vestibular cranial nerves are exceedingly rare in non-syndromic patients but are encountered in approximately 50% of NF2 patients. The most common sites of non-vestibular schwannomas in NF2 are the trigeminal nerves (Fig. 3a), followed by the oculomotor nerves (Fig. 3b) [51]. Lesions may be diagnosed in other sites (Fig. 3c) and have been described involving each of the cranial nerves. Schwannomas also frequently arise from spinal nerve roots and peripheral nerves in NF2 patients [52]. Plexiform schwannomas which involve multiple nerves are often encountered in patients with NF2, predominantly in the cervical region (Figs. 3a, 4d–f), brachial plexus (Fig. 3d), and sacral plexus (Fig. 3e, f) [53]. Such lesions may have different genetic alterations in each lobule of a mass, suggestive of a polyclonal origin [54]. Distinctive plexiform “plaque-like” dermal or subcutaneous schwannomas (Figs. 3g, 4a) may be encountered in NF2 which are not seen in the sporadic setting or schwannomatosis [52]. Such lesions consist of neoplastic Schwann cells which expand peripheral nerves and infiltrate the adjacent dermis and subcutis as well as surround adnexal structures such as hair follicles, sebaceous glands (Fig. 4b), and eccrine glands (Fig. 4c). Other unusual sites may also be involved including intramuscular schwannomas (Fig. 3h) and infiltrative intra-tendinous schwannomas (Fig. 3i). NF2-associated tumors more frequently infiltrate nerve fibers (Fig. 4g–j) compared to sporadic lesions which more frequently grow adjacent to and compress the nerve [55]. Like sporadic schwannomas, each of these lesions exhibits strong and diffuse staining with S100 by immunohistochemistry. Recently, it was found that immunohistochemistry for INI1/SMARCB1 demonstrates a mosaic pattern of loss in NF2-associated tumors. Such findings may also raise the possibility of schwannomatosis, and this possibility should be considered in the absence of genetic testing or definitive NF2 diagnosis [56].

Schwann cell tumorlets are often observed studding the spinal nerve roots of patients with NF2 (Fig. 4k, l). These lesions may show loss of *NF2* gene function and are hypothesized to represent precursor lesions to schwannomas [57]. NF2 patients may also develop ‘schwannosis’, characterized by proliferations of neoplastic cells without frank mass formation, typically along the dorsal root entry zones of the spine. Interestingly, whole genome methylation studies showed distinct clustering of vestibular and peripheral schwannomas, suggesting that they have unique biological features [58].

Although multiple schwannomas are observed in both NF2 and schwannomatosis, the clinical features of the two syndromes are distinct. NF2-associated schwannomas are often present early in childhood or young adulthood and are often associated with neurologic deficits such as weakness or sensory loss, while schwannomatosis-associated lesions are more often diagnosed later in life and are more frequently associated with pain [59]. Histologically, schwannomatosis-associated lesions may also exhibit unusual growth patterns like NF2-associated lesions, including infiltrative intraneural growth, and myxoid changes [13], though cutaneous “plaque-like” schwannomas are not seen. Though it was initially suspected that malignant peripheral nerve sheath tumors (MPNST) may be more common in NF2 patients, a study of a large cohort of NF2 patients showed no convincing evidence for an increased risk of malignant transformation of schwannomas in non-irradiated patients [60].

Immunostaining of a cohort of NF2-associated schwannomas showed VEGF expression in 100% of vestibular schwannomas and VEGFR-2 in 32% of tumor vessels. Treatment with the anti-VEGF agent bevacizumab in a retrospective study of ten consecutive patients with NF2 and growing vestibular schwannomas showed reduction of tumor volume and improved hearing in some of the patients [61]. Anti-VEGF treatment in combination with radiation has also shown efficacy in some studies in pre-clinical trials and decreased the growth rate of vestibular schwannomas in human studies [62, 63]. Anti-VEGF treatment may be particularly useful in cases in which surgery is not possible.

Immunohistochemistry in both NF2-associated and sporadic human schwannomas shows evidence of activated MAPK signaling including increased phospho-AKT, phospho-MEK, and phospho-ERK, as well as increased nuclear c-JUN [64]. These changes are presumed to result from loss of merlin protein and dysregulation of MAPK and RAS signaling. In support of this hypothesis, over-expression of merlin in RAS-driven schwannoma cell lines can suppress growth in a density-dependent fashion, and these effects seem to result from reduction in downstream MEK–ERK signaling [65]. Several MEK inhibitors are under investigation for the treatment of NF2-associated schwannomas and these agents have demonstrated some efficacy at reducing growth in mouse models and in vitro studies [66–68]. Given the success of pre-clinical studies, the MEK inhibitors selumetinib and cobimetinib are being explored in human clinical trials for treatment of NF2-associated tumors (NCT02639546, NCT03095248). mTOR signaling has also been experimentally demonstrated to be dysregulated in NF2-associated schwannomas, and clinical trials with mTOR inhibitors such as everolimus and sirolimus have shown efficacy in reducing the growth of tumors in NF2 patients [69, 70].

Additional putative targets for therapeutic intervention continue to be identified due to increasing understanding of NF2 biology. One novel target may be the membrane receptor CXCR4, which exhibited increased expression in vestibular schwannomas by IHC and mRNA profiling [71]. The receptor tyrosine kinase cMET was found to have increased expression in some cases in schwannoma mouse models and organoid slices, and expression of this protein correlated with increased tumor growth. Inhibition of cMET reduced tumor growth and sensitized schwannomas to radiation in mouse models, providing one potential avenue to reduce radiotoxicity in NF2 patients [72]. More recently, substantial interest has been focused on immunotherapeutic avenues including checkpoint inhibition with PD-1 and PD-L1 inhibitors due to the remarkable efficacy of these agents in a wide variety of tumors. Evaluation of NF2-associated tumors has been limited to date, but one study showed PD-L1 expression (> 5% of tumor cells) in a substantial subset of NF2-associated schwannomas, suggesting that this may be an interesting avenue for further clinical study [73]. Inhibition of EGFR signaling via lapatinib or nilotinib, particularly in combination with radiation therapy, showed efficacy in NF2-deficient mouse models suggesting this as a further avenue for therapeutic study [74]. A subset of NF2-associated schwannomas may also harbor the *SH3PXD2A*-*HTRA1* gene fusion that has been identified in sporadic schwannoma [75].

Despite these advances, no trial-proven medical therapies have been FDA approved to date for NF2-associated schwannomas. In addition to ongoing clinical trials, further analysis of molecular data, including genomic and epigenomic studies in larger cohorts of patients, may identify additional putative targets for therapeutic intervention.

### Meningioma

**Fig. 5:**
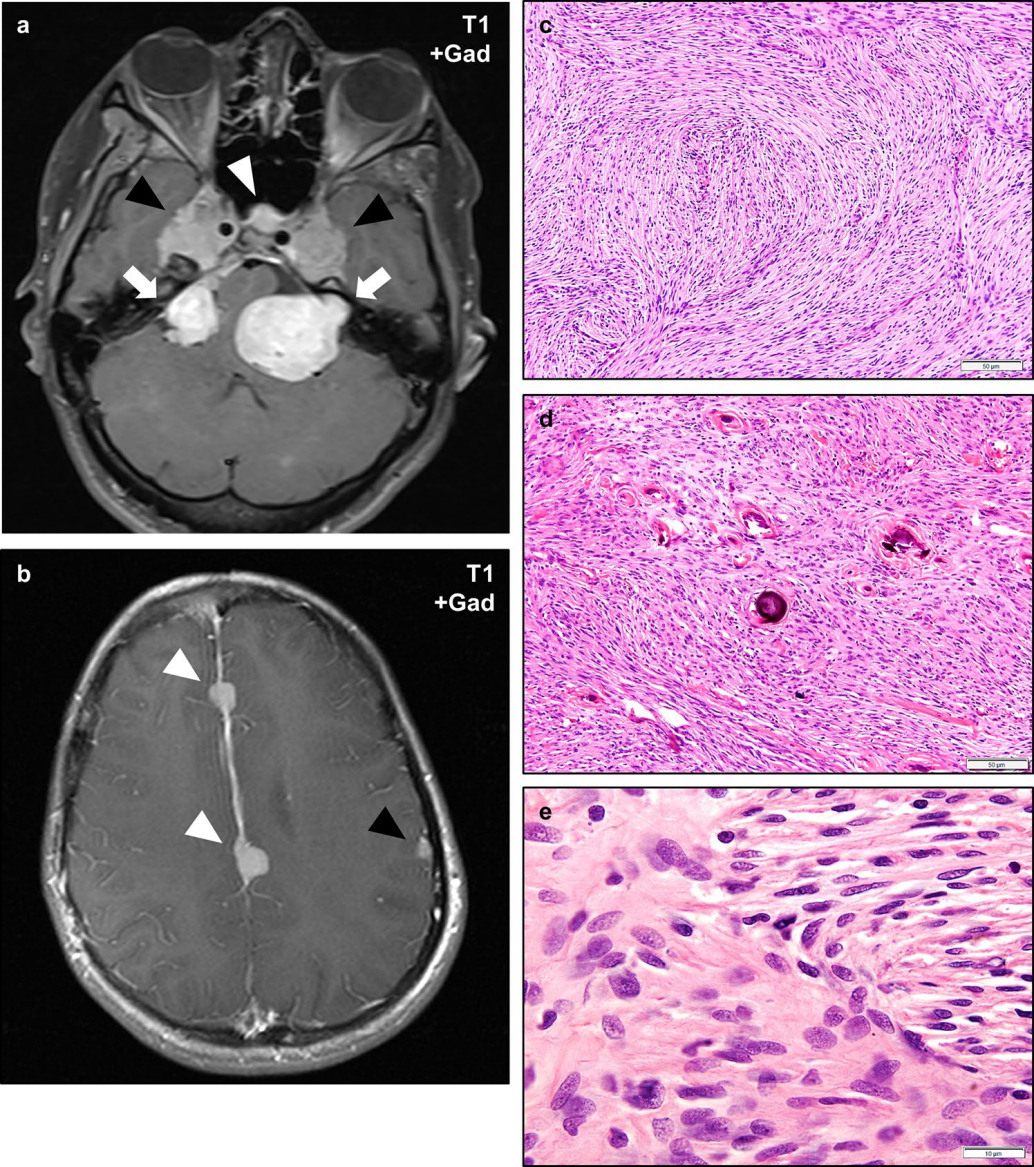
Meningiomas in NF2. NF2 patients frequently develop multiple meningiomas, which may involve unusual anatomic locations. Post-gadolinium T1-weight magnetic resonance imaging of the skull base of an NF2 patient with bilateral vestibular schwannomas (**a**, arrows) and durally based masses consistent with meningiomas in the bilateral Meckel’s caves (black arrowheads) and cavernous sinus (white arrowhead). This patient also had multiple meningiomas involving the falx cerebri (white arrowheads) and cerebral convexity (black arrowhead) (**b**). Histologically, NF2-associated meningiomas most often exhibit fibrous morphology (**c**), though any histologic pattern may be encountered. A second NF2-associated meningioma shows scattered psammoma bodies (**d**). The cells of NF2-associated schwannomas are cytologically similar to sporadic meningiomas and most often exhibit ovoid to spindled cells with find chromatin, scattered nuclear pseudo-inclusions, and mild cytologic atypia (**e**). Scale bars 50 μm (**c**, d), 10 μm (**e**)

Meningiomas are proliferations of neoplastic cells with histologic, ultrastructural, and immunophenotypic evidence of meningothelial cell differentiation which have a wide variety of histologic appearances and genetic aberrations. Intracranial meningiomas are diagnosed in 45–58% of individuals with NF2, and spinal meningiomas are diagnosed in approximately 20% [76]. NF2-associated meningiomas occur most frequently in the supratentorial region in the frontal, parietal, and temporal regions as well as along the falx cerebri (Fig. 5a, b). However, they may occur anywhere in the central nervous system including the spinal canal and unusual locations such as the cerebral ventricles and optic nerve sheaths. NF2-associated meningiomas appear to arise less frequently at locations in the anterior and middle skull base [77], similar to sporadic *NF2*-mutated meningiomas which have a predilection for the dura overlying the cerebral convexities [78].

NF2-associated meningiomas may be of any histologic subtype, but are most often of the fibrous variant (Fig. 5c–e) [79]. Similarly, sporadic meningiomas with *NF2* inactivation also tend to be of the fibrous subtype [78, 80]. The biological basis for the development of fibrous morphology in NF2-deficient tumors is not entirely clear, but it is interesting to speculate whether it may be related to dysregulation of contact inhibition and cell–cell adhesion due to merlin deficiency, with resultant development of a more ‘mesenchymal' spindled phenotype. NF2-associated tumors frequently exhibit a higher mitotic index than histologically comparable sporadic tumors, and atypical meningiomas are not uncommonly encountered in this setting [81]. The genetic basis for these differences is not entirely clear, as sporadic grade I meningiomas also frequently exhibit monosomy 22 and loss of function of *NF2.*

The occurrence of multiple meningiomas is a hallmark of NF2 and a major diagnostic criterion for the syndrome, occurring in about 50% of patients with NF2. Examination of large patient populations shows that the presence of multiple meningiomas, particularly in pediatric patients, is superior to the presence of a unilateral vestibular schwannoma for the diagnosis of NF2 [44]. While only approximately 5% of patients with a meningioma in the general population will have multiple lesions, 20% of such patients are diagnosed with NF2. Rarely, the presence of multiple meningiomas may be associated with other syndromes resulting from alterations in *SUFU* [82], *SMARCE1* [83], and *SMARCB1* (schwannomatosis) [84]. *SMARCB1*-associated lesions may be particularly problematic, although typically bilateral vestibular schwannomas and ependymomas are not typically identified in schwannomatosis patients. NF2-associated meningiomas frequently occur at an earlier age than sporadic tumors, and presentation with meningioma in the pediatric setting should raise suspicion for NF2 [81].

Systematic genetic profiling of NF2-associated meningiomas has not yet been completed, but limited studies suggest that, like sporadic tumors, atypical tumors are associated with an increased burden of chromosomal copy-number changes and may exhibit deleterious gene alterations in addition to *NF2* loss [85]. Epigenomic profiling may provide additional insights into the biology of NF2-associated lesions.

Loss of merlin function in sporadic or NF2-associated meningiomas may result in abnormal activation of mTOR signaling [86], and inhibition of this pathway via rapamycin resulted in slowed growth of tumors in experimental settings. A clinical trial with the mTOR inhibitor RAD001 also showed a similar effect at reducing growth, though tumor volume was not reduced from baseline in either study [87]. An additional trial with the mTORC1/2 inhibitor AZD2014 is also ongoing in meningioma (NCT02831257, NCT03071874). Screening for additional targets suggested that co-inhibition of EPHA2 may further reduce proliferation of NF2-associated meningiomas in combination with mTORC1/2 inhibition [88], though this finding requires further clinical validation.

Merlin-deficient cells have been found to demonstrate sensitivity to inhibitors of focal adhesion kinase (FAK) inhibition [89, 90]. It is hypothesized that this potential therapeutic vulnerability in merlin-negative cells, such as those of malignant mesothelioma and high-grade serous ovarian cancer, is due to a greater dependence on cell–extracellular matrix-induced FAK signaling within these cells. Recently, phase I studies of FAK inhibitors (GSK2256098 and VS-6063) in patients with advanced solid tumors showed clinical activity in patients with mesothelioma, with one study suggesting particularly enhanced activity in patients with mesothelioma lacking merlin [91, 92]. Inhibiting FAK with GSK2256098 is currently under investigation in a phase 2 trial for patients with NF2-altered meningioma (NCT02523014/A071401). Additional large-scale drug screening studies are underway for NF2-associated meningioma and schwannoma to identify additional therapeutic targets [93].

### Ependymoma

**Fig. 6:**
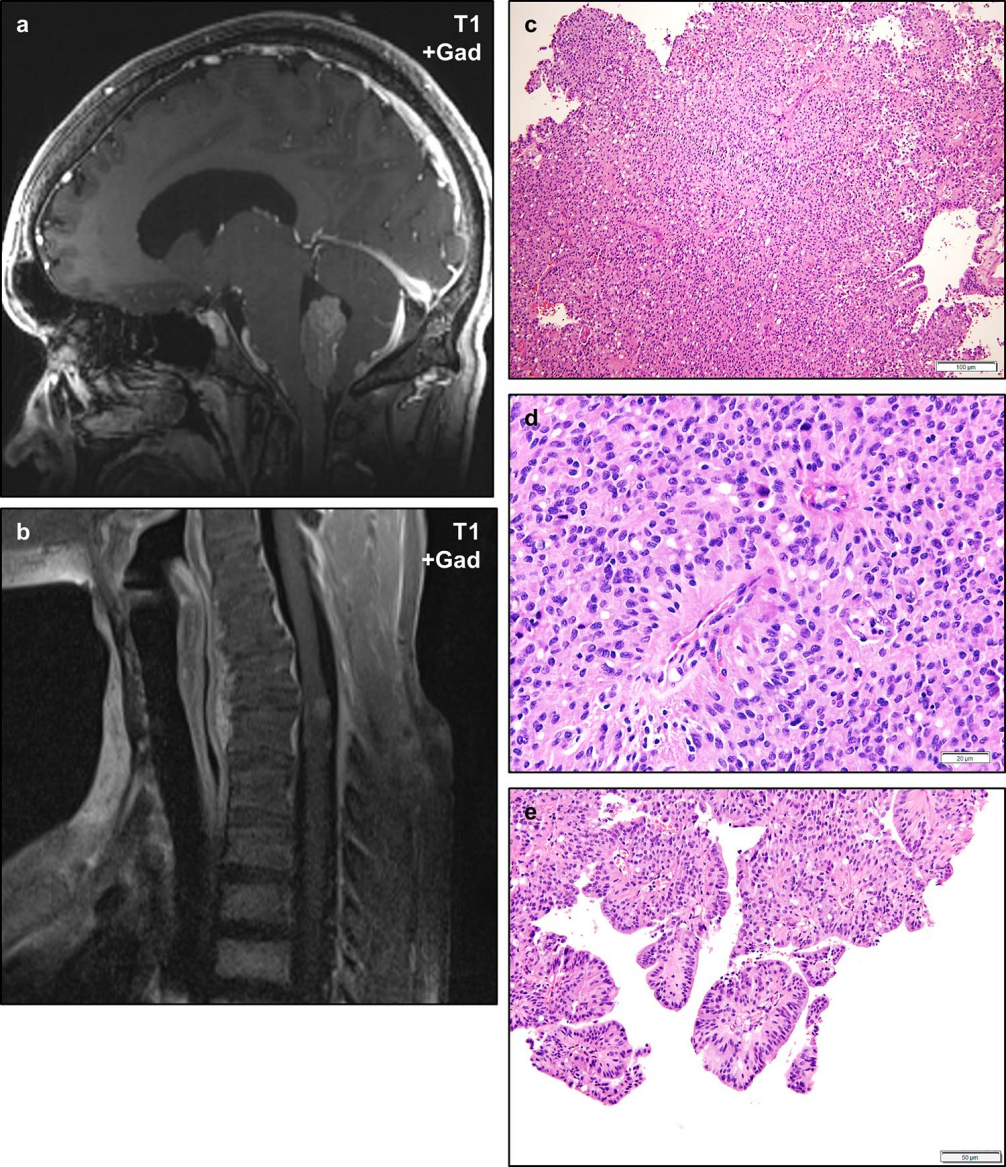
Ependymomas in NF2. Ependymomas are frequently diagnosed in NF2 patients and may occur in a variety of locations including unusual sites. Post-gadolinium T1-weighted magnetic resonance imaging of an NF2 patient shows a weakly enhancing intraventricular mass in the fourth ventricle (**a**). A second NF2 patient developed an intramedullary mass in the cervical spine (**b**). Histologically, the fourth ventricular mass exhibited typical features of ependymoma including prominent perivascular pseudorosettes and scattered true ependymal rosettes (**c**, **d**), with some regions exhibiting prominent papillary architecture (**e**). Scale bars 100 μm (**c**), 20 μm (**d**), 50 μm (**e**)

Ependymomas are proliferations of neoplastic cells with histologic, molecular, and ultrastructural similarities to the ependymal cells of the ventricles and spinal canal. Ependymomas are diagnosed in approximately 33–53% of individuals with NF2, most commonly involving the posterior fossa and cervicomedullary region of the spine (Fig. 6a, b) [94]. The majority of NF2-associated ependymomas are WHO grade II lesions with classic histology including perivascular pseudorosettes and ependymal rosettes (Fig. 6c, d), though features such as papillary architecture (Fig. 6e) and other histologic subtypes including myxopapillary, tanycytic, and anaplastic ependymomas may be observed. NF2 patients frequently develop multiple spinal ependymomas, which may assume a characteristic ‘string of pearls’ appearance along the spinal cord and cauda equina. NF2-associated ependymomas more often arise in patients with truncating *NF2* mutations, suggesting that ependymomas are associated with more aggressive variants of the syndrome, as with meningiomas.

Interestingly, pathologic review has shown that many lesions initially classified as “gliomas” by the Manchester classification system are in fact ependymomas, and not diffuse or piloid gliomas [95]. Revision of the classification system to replace the term ‘glioma’ with ependymoma has been proposed, and study of a large patient population demonstrated that this change would improve diagnostic yield [44].

NF2-associated spinal ependymomas are frequently treated conservatively due to an absence of symptoms in most cases, and the risk of morbidity associated with resection, though surgery may be necessary and beneficial in some patients [96]. Trials of systemic therapeutic intervention in NF2-associated ependymomas are limited given their typical conservative management. Small cohorts of patients treated with bevacizumab have shown improvement in symptoms and limited efficacy in reducing tumor size and growth in a subset of NF2 patients, though further studies in large randomized cohorts are needed [97]. Burgeoning molecular analysis of ependymal lesions in NF2 patients may provide novel molecular targets for clinical investigation.

### Glioma

Diagnosis of a glioma has traditionally been used as a diagnostic criterion for NF2; however, on clinicopathologic review nearly 80% of lesions characterized as NF2-associated gliomas are found to be spinal intramedullary or cauda equina ependymomas. Diffuse astrocytomas and pilocytic astrocytomas are uncommonly observed in patients with NF2, but the causal association of these lesions with NF2 is unclear. Clinicopathologic studies have shown no definite evidence of association between high-grade gliomas and NF2, though these lesions may arise secondarily in NF2 patients, often in those who receive radiation therapy for other syndrome-associated masses such as meningiomas [98]. Interestingly, *NF2* alterations are often observed in diffuse gliomas, though typically as sporadic alterations, and NF2 does not seem to significantly predispose to the development of such lesions. Given that most NF2-associated gliomas are in fact ependymomas, revision of the Manchester classification system to replace the term ‘glioma’ as a diagnostic criterion with ‘ependymoma’ has been suggested, though such changes have not yet been adopted. Analysis of a large patient cohort showed that inclusion of the broader term ‘glioma’ encompassing both gliomas and ependymomas in the 2017 revised Manchester classification provided no improvement in positive predictive value compared to ependymoma alone [44].

### Meningioangiomatosis

**Fig. 7:**
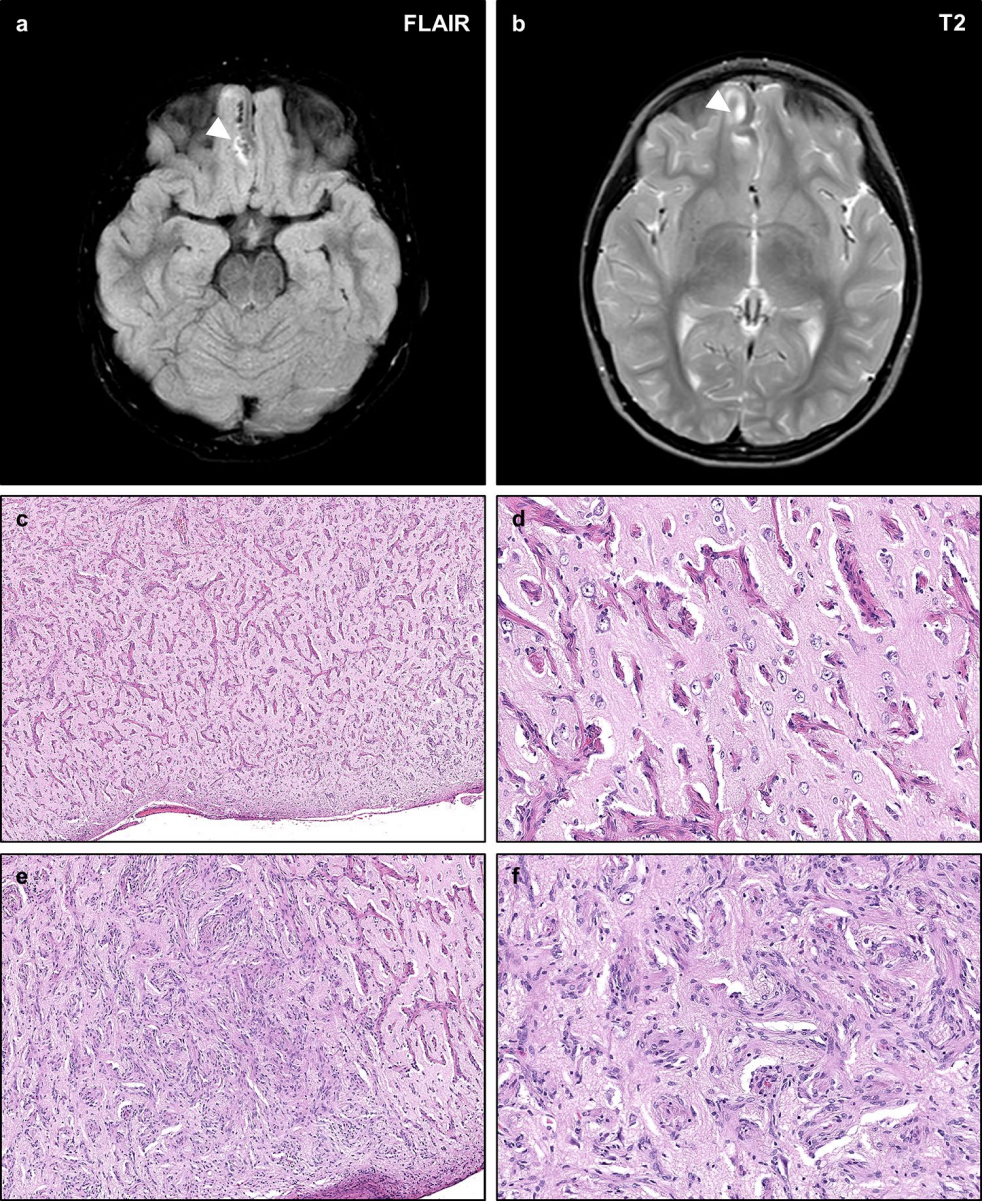
NF2-associated meningioangiomatosis. Meningioangiomatosis is a rare plaque-like leptomeningeal and perivascular proliferation of fibroblastic and meningothelial-appearing cells which may extend along Virchow–Robin spaces. Such lesions are typically hyperintense on FLAIR (**a**) and T2-weighted sequences (**b**), with intense contrast enhancement on post-gadolinium T1-weighted sequences. Histologic examination of this lesion from an NF2 patient with refractory epilepsy shows a mixed perivascular proliferation of fibrovascular (**c**, **d**) or meningothelial-like cells (**e**, **f**) extending from the pial surface into the cortical parenchyma. The intervening cortex shows intact neurons with no definite evidence of focal cortical dysplasia. The neoplastic cells are typically well differentiated with no necrosis, atypia, or prominent nucleoli

Meningioangiomatosis is rare poorly understood lesion, with approximately 200 cases described in the literature, which has been associated with NF2 [99]. Lesions are typically characterized by a plaque-like leptomeningeal and perivascular proliferation of small blood vessels, meningothelial-like, and fibroblastic cells with focal hyalinization and calcification which may extend into Virchow–Robin spaces and along small intra-parenchymal vessels (Fig. 7c–f) [100, 101]. The proportion of each component varies, and lesions may be predominantly comprised of vascular structures, resulting in lesions that resemble vascular malformations, or predominantly meningothelial and fibroblastic cells in more cellular lesions. Calcifications, psammoma bodies, and meningothelial whorls may be present. The mitotic rate and ki-67 index (< 2%) are typically low, and necrosis, nuclear atypia, and nucleolar enlargement are not observed. The lesions are most often associated with the cortical surface, but non-cortically based lesions within deep structures have been described [102]. Surrounding brain parenchyma may show a variety of changes including meningeal hyperplasia and focal cortical dysplasia in cortically based lesions, and perivascular white matter edema, gliosis, and sclerosis in subcortical lesions.

Meningioangiomatosis was first described by Bassoe and Nuzum in 1915 in association with neurofibromatosis and was initially thought to be associated with NF1 [103]. Subsequent studies showed that meningioangiomatosis was instead associated with NF2 [104]. Though most cases are now recognized to be sporadic, a substantial proportion of cases (12–35%) are associated with syndromic NF2. Interestingly, despite the strong association of meningioangiomatosis and NF2, and the presence of germline *NF2* mutations in cases of NF-associated meningioangiomatosis, sporadic lesions do not typically demonstrate mutations in *NF2* [104, 105]. The genetic basis of these lesions is presently not clear. Sporadic lesions most often present with seizures (73% of cases), followed by headaches as the second most common presentation [106]. However, a variety of other presentations may be observed depending on the anatomic distribution of disease, including cortical blindness, paresis, or behavioral changes [99]. More severe presentations including sudden death related to epilepsy [107] or intracerebral hemorrhage [108] have been reported, suggesting that careful clinical monitoring is warranted following diagnosis. Interestingly, in contrast to sporadic lesions, NF2-associated lesions are often asymptomatic. The biological basis for differences in clinical presentation between sporadic and NF2-associated lesions is not known.

Meningioangiomatosis may present as a solitary lesion, or as a multi-focal or diffuse lesion. Multi-focal presentation is more common in NF2-associated lesions (35% vs. 13% of sporadic lesions), as with other NF2-associated lesions such as schwannomas and meningiomas. In sporadic cases, the frontal lobe is the most common location, followed by the temporal, parietal, and occipital lobes. In contrast, NF2-associated cases are most frequently located in the temporal lobe, followed by the parietal lobe. There may be a slight right-sided predisposition for both sporadic and NF-associated lesions. Lesions are only rarely primarily located in deep subcortical regions or the posterior fossa. While most sporadic lesions are unifocal, approximately 50% of NF2-associated lesions are multi-focal at presentation, and more frequently involve unusual sites such as the posterior fossa or deep subcortical structures. The biological basis for these differences in anatomic distribution is unclear but may provide an opportunity for further research.

Radiologically, meningioangiomatosis typically presents as a hypodense lesion on CT imaging, usually with heterogeneous contrast enhancement. Calcifications are present in approximately 90% of cases and may be helpful in the diagnosis. Lesions are hypointense to isointense on T1 MRI sequences, and hyperintense on FLAIR (Fig. 7a) and T2 (Fig. 7b) sequences, frequently with a gyriform pattern reflecting their leptomeningeal pattern of spread. Lesions most often show heterogeneous enhancement with gadolinium, likely as a result of varying degrees of vascular cells in different regions of the lesions [109–111]. Cerebral angiograms are typically normal and not useful for the diagnosis, only rarely showing abnormal vessels. Lesions are bright by PET imaging, which may be helpful for defining their distribution [112].

Meningioangiomatosis was traditionally considered to represent a benign hamartomatous lesion; however, increasing evidence suggests that it may represent a pre-neoplastic or neoplastic entity. Meningioangiomatosis may be associated with meningiomas, most often of the fibrous or transitional subtypes. In such cases, there is typically no clear border between the two lesions, and the cells of the meningioangiomatosis often histologically resemble those of the adjacent meningioma. Genetic analyses have demonstrated that both the meningioma and associated meningioangiomatosis frequently share genetic alterations, strongly supporting a clonal origin of both lesions in at least some cases. While invasion of brain parenchyma by meningiomas portends a worse prognosis and can be considered an independent criterion for diagnosis of atypical meningioma (WHO grade II) by 2016 criteria [1], cortical or deep perivascular spread in a meningioangiomatosis pattern is not yet associated with WHO grading or prognosis. However, some evidence suggests that meningioma-associated meningioangiomatosis is associated with increased risk of meningioma recurrence.

Meningioangiomatosis may rarely be associated with malignant sarcomatous lesions. In at least one case report, a patient developed a malignant SMARCB1-deficient desmoplastic spindle cell sarcoma associated with pre-existing sporadic meningioangiomatosis in the absence of germline *SMARCB1* or *NF2* mutations. Immunophenotypically, the meningioangiomatosis component in these cases exhibited staining with SMARCB1, but this expression was lost in the dedifferentiated sarcomatous component. Molecular analyses of the meningioangiomatosis and sarcoma in this case showed that the meningioangiomatosis exhibited multiple recurrent chromosomal alterations including monosomy 22, 6q gain, and 5q loss. Interestingly, monosomy 22 and 6q gain were also observed in the sarcomatous lesion, in addition to numerous other chromosomal alterations, suggesting that the tumor may have arisen from the underlying meningioangiomatosis through acquisition of additional chromosomal alterations and silencing of SMARCB1 expression [113].

### Glial micro-hamartomas (hamartia)

**Fig. 8:**
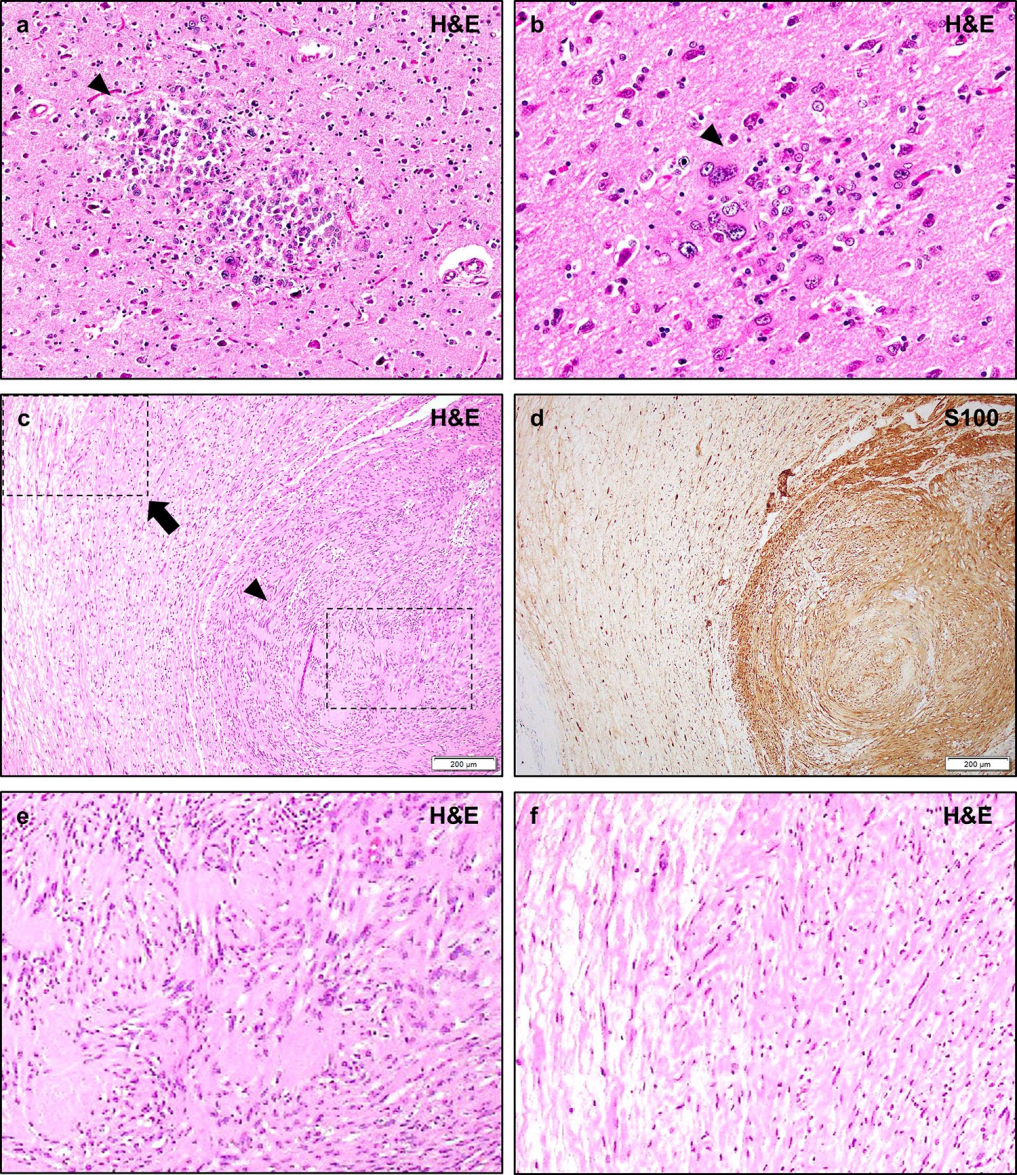
Other lesions in NF2. Post-mortem examination of NF2 patients may demonstrate small circumscribed collections of immature-appearing glial cells known as glial hamartia (**a**, **b**, arrowheads). The cells of these lesions typically exhibit eosinophilic cytoplasm, pleomorphic nuclei, and occasional multi-nucleation. The cells are strongly S100 positive, with occasional focal GFAP staining, but their precise lineage derivation is not certain. The surrounding cortex may exhibit reactive changes but does not typically exhibit features of focal cortical dysplasia. Hybrid schwannoma/neurofibromas are frequently encountered in NF2 patients (**c**, **d**), and typically exhibit nodular regions of schwannoma with typical histologic features such as Verocay bodies and hyalinized vessels, a monomorphic cellular population, and strong/diffuse S100 staining (**e**, arrowhead), while other regions exhibit lower density regions with mixed cellularity, loose “shredded carrot” collagen, and less prominent S100 staining consistent with a neurofibroma component (**f**, arrow). While pure neurofibromas are occasionally encountered in NF2 patients, they are typically sporadic solitary masses, in contrast to the multiple plexiform neurofibromas of NF1. Pathologic review shows that many lesions initially diagnosed as neurofibromas in NF2 patients are in fact misdiagnosed hybrid schwannoma/neurofibromas. Scale bars 200 μm (**c**, **d**)

Glial micro-hamartomas are circumscribed collections of immature-appearing glial cells with atypical pleomorphic nuclei, occasional multi-nucleation, eosinophilic cytoplasm (Fig. 8a, b), and an abnormal immunophenotypic profile characterized by strong S100 immunoreactivity, occasional focal staining with GFAP [1, 114], and negative staining for other lineage (endothelial, mesenchymal, neural, myogenic) markers. Ultrastructurally, the atypical cells demonstrate no evidence of ependymal or neural differentiation such as multi-ciliation or neurosecretory granules. The surrounding cortex may show some minimal disruption of immediately adjacent myelinated fibers, but the cortex is typically unremarkable without the characteristic cytologic or architectural features of focal cortical dysplasia.

Post-mortem examination of the brains of patients with NF2 shows that such lesions are common and scattered throughout the cerebral gray matter in all regions, most commonly in the molecular and deep cortical layers. Micro-hamartomas are also occasionally observed in other regions of the brain including the basal ganglia, thalamus, cerebellum, brainstem, and spinal cord. The presence of scattered glial micro-hamartomas is essentially pathognomonic of NF2, and they are found in most cases with rigorous examination [102]. Glial micro-hamartomas are apparently asymptomatic, and their clinical significance in NF2 patients is not well understood. They are not known to be associated with cognitive deficits or seizures, but further investigation of potential functional implications of these lesions in NF2 patients may provide interesting insights.

Radiologically, glial micro-hamartomas may be visible as hyperintense regions on T2 or fluid-attenuated inversion recovery (FLAIR) sequences. The radiologic appearance is similar to and frequently indistinguishable from focal cortical dysplasia, and a “transmantle sign” may be present with tapering of signal abnormalities from the cortical surface to the ventricle suggestive of a possible defect in tangential migration, though as noted definitive histologic features of focal cortical dysplasia are typically not present [115]. Despite the strong association of such lesions with NF2 and their frequent presence in post-mortem NF2 brain specimens, a lack of specificity in their imaging appearance hampers their use as a clinical diagnostic criterion, though advances in radiologic assessment may provide opportunities for exploiting this common feature of NF2 diagnostically.

Despite their atypical cytologic appearance, such collections of cells do not reach macroscopic size, and have not been found in association with gliomas. Given their association with other malformative lesions in NF2 and absence of apparent growth potential, such collections of cells are presumed to represent benign hamartomatous lesions. Molecular analyses have demonstrated that merlin expression is retained and may even be accentuated in the atypical cells of glial micro-hamartomas [116]. It has not been rigorously determined whether this expression of merlin represents wild-type or mutant protein; however, these findings suggest that glial micro-hamartomas may result from haploinsufficiency of merlin or dominant-negative mutant protein function rather than complete loss of expression within lesional cells. Nevertheless, the precise lineage differentiation of the cells in glial micro-hamartomas and their biological basis remains unclear.

### Neurofibroma

Despite its nomenclature as a neurofibromatosis syndrome, NF2 only rarely presents with pure neurofibromas. Plexiform lesions in patients with NF2 are typically revealed to represent plexiform schwannomas, in contrast to the plexiform neurofibromas of NF1. Interestingly, on pathologic review, up to 26% of nerve sheath tumors in NF2 exhibit features of both neurofibroma and schwannoma, and most lesions diagnosed as neurofibroma are more appropriately classified as hybrid neurofibroma/schwannomas (Fig. 8c–f) [117, 118]. Immunohistochemistry may be useful in the classification of such lesions, typically showing nodular schwannoma regions with stronger and more diffuse S100 staining (Fig. 8d). The value of continued inclusion of neurofibromas in the Manchester classification system (Table 1) is uncertain, and recent studies have suggested removal of this entity as a diagnostic feature of NF2 [44].

## Other manifestations of neurofibromatosis type II

### Intracranial calcifications

Some imaging studies of patients with NF2 have disclosed focal T1/T2 hypointense regions suggestive of mineralization in a subset of patients, most frequently in the superficial cerebral and cerebellar cortices [115]. However, many such lesions may be associated with underlying intracranial meningiomas or meningioangiomatosis, each of which often presents with calcifications. The pathologic basis of this radiologic finding in many patients is not clear and presents an opportunity for further study.

### Peripheral neuropathy

Epidemiologic studies have revealed that NF2-patients frequently present with peripheral neuropathies. The presentation is most often as a mononeuropathy in children, and as a polyneuropathy in adults. Nerve biopsies from patients with NF2 suggest that this may result from local compression of nerves by Schwann cell tumorlets or non-mass forming Schwann cell proliferations. Whether intrinsic dysfunction of non-neoplastic Schwann cells also plays a role is largely unclear. Interestingly, several studies showed impaired recovery of peripheral nerve function and myelination following crush injury in transgenic mice with NF2-deficiency localized to Schwann cells in the absence of neoplastic lesions, suggesting that non-neoplastic Schwann cells may also have defective cellular homeostasis in NF2 patients [119, 120]. Nevertheless, the effects of merlin haploinsufficiency in non-neoplastic Schwann cells in NF2 patients remains poorly understood, and an intriguing area for further investigation.

### Ophthalmologic lesions

Ophthalmologic lesions are present in most patients with NF2 [121]. The most common lesions are cataracts resulting from posterior subcapsular lenticular opacities, which are present in approximately 60–80% of NF2 patients [52]. Juvenile cortical wedge cataracts may be observed shortly after birth in patients with NF2, with posterior subcapsular opacities developing later in life. NF2 patients may also develop retinal hamartomas and optic nerve sheath meningiomas which may affect vision [122]. This may be particularly problematic if such lesions are misdiagnosed clinically as retinoblastomas. The biological basis for NF2-associated cataracts and subcapsular opacities is poorly understood, though they presumably result from loss of merlin and dysfunction of intracellular signaling in these cellular populations, though this requires further analysis.

### Cutaneous lesions

NF2 patients frequently manifest with cutaneous lesions, though these lesions are typically far less prominent than those of NF1 patients. Approximately 70% of NF2 patients exhibit cutaneous lesions, with > 10 lesions identified in 10% of patients. Most frequent are “plaque-like” dermal plexiform schwannomas associated with slight pigmentation and increased hair growth. Subcutaneous nodular schwannomas associated with peripheral nerves are also identified. Rarely, neurofibromas may be encountered, though these do not seem to be a characteristic feature of the disease despite the nomenclature and are often found to be more appropriately classified as hybrid neurofibroma/schwannomas on pathologic review. The presence of dermal and cutaneous plexiform schwannomas is common and may occur early in the disease course, particularly in patients with the more severe Wishart phenotype, allowing for earlier clinical recognition of the syndrome, genetic analysis, and diagnosis [115].

## Conclusion

Neurofibromatosis type II is a familial tumor predisposition syndrome characterized by the development of distinctive nervous system lesions including bilateral vestibular schwannomas, multiple spinal and peripheral schwannomas, meningiomas, and ependymomas. NF2 results from alterations in the *NF2* gene on chromosome 22, with resultant dysfunction of its ERM-family protein product merlin. Systematic analysis of merlin function has shown interactions with numerous intracellular signaling pathways, with tumorigenesis likely resulting from complex lineage-specific mechanisms. However, dysfunction of merlin-mediated contact growth inhibition is hypothesized to play a central role in multiple tumor types. Dysregulation of RAS–MEK–ERK and mTORC1/2 signaling has been shown to promote tumorigenesis in NF2-associated schwannomas and meningiomas, and inhibition of these pathways has shown promise in multiple therapeutic trials in NF2 patients. Multiple additional pathways have been identified, and pre-clinical and clinical investigation of novel therapeutic agents including FAK, EGFR, cMET, PD-1/PD-L1, and VEGFR inhibitors is underway.

Though NF2 was recognized as a distinct genetic syndrome in the 1980s and abundant descriptions of the disease have been reported since that time, clinicopathologic studies continue to refine the diagnostic criteria of NF2 and suggest novel associated lesions such as meningioangiomatosis and glial hamartia. At the same time, inclusion of neurofibromas as a diagnostic criterion in NF2 is of questionable significance given the poor association of these lesions with the disease despite its nomenclature as a neurofibromatosis. The diagnostic term ‘glioma’ may also be too broad and require further refinement, as diffuse gliomas are not strongly associated with the disease, and many lesions classified as such in NF2 patients are revealed to be ependymomas on review. Ongoing integration of molecular and clinicopathologic features is likely to provide further refinements to the diagnostic framework of NF2. Efforts are also underway to identify biochemical and molecular biomarkers useful for the diagnosis, prognostic evaluation, and management of patients with NF2.

Genome-wide genetic and epigenetic studies have revealed significant insights into the biology of NF2, and analysis of larger patient cohorts is likely to further increase understanding of the molecular biology of NF2-associated tumors and suggest additional therapeutic avenues for clinical interrogation. Moreover, insights derived from study of merlin have yielded fundamental insights into regulation of intracellular signaling pathways, which may have applications in other disease and biological systems. In this sense, NF2 continues to demonstrate how systematic molecular and clinicopathologic study of tumor predisposition syndromes may yield substantial biological insights in addition to improvement in clinical care.
