# Correction to: An update on the CNS manifestations of neurofibromatosis type 2

**DOI:** 10.1007/s00401-019-02044-6

**Published:** 2019-08-20

**Authors:** Shannon Coy, Rumana Rashid, Anat Stemmer-Rachamimov, Sandro Santagata

**Affiliations:** 1 Division of Neuropathology, Department of Pathology, Brigham and Women’s Hospital, Hale Building for Transformative Medicine, BTM8002P, 60 Fenwood Road, Boston, MA 02115 USA; 2 Department of Pathology, Boston Children’s Hospital, Boston, MA USA; 3 Harvard Medical School, Boston, MA USA; 4 Laboratory for Systems Pharmacology, Harvard Program in Therapeutic Science, Boston, MA USA; 5 Department of Pathology, Massachusetts General Hospital, Boston, MA USA; 6 Ludwig Center at Harvard, Boston, MA USA

## Correction to: Acta Neuropathologica 10.1007/s00401-019-02029-5

The article An update on the CNS manifestations of neurofibromatosis type 2, written by Shannon Coy, Rumana Rashid, Anat Stemmer-Rachamimov and Sandro Santagata, was originally published electronically on the publisher’s internet portal (currently SpringerLink) on 04 June 2019 without open access.

With the author(s)’ decision to opt for Open Choice the copyright of the article changed on [20 August 2019] to © The Author(s) 2019 and the article is distributed under the terms of the Creative Commons Attribution 4.0 International License (http://creativecommons.org/licenses/by/4.0/), which permits unrestricted use, distribution, and reproduction in any medium, provided you give appropriate credit to the original author(s) and the source, provide a link to the Creative Commons license, and indicate if changes were made.

The original article has been corrected.

